# Current Status and Molecular Mechanisms of Resistance to Immunotherapy in Oral Malignant Melanoma

**DOI:** 10.3390/ijms242417282

**Published:** 2023-12-08

**Authors:** Sena Zeynep Usta, Toshihiro Uchihashi, Shingo Kodama, Kyoko Kurioka, Toshihiro Inubushi, Takuya Shimooka, Akinari Sugauchi, Soju Seki, Susumu Tanaka

**Affiliations:** 1 Department of Oral and Maxillofacial Surgery, Graduate School of Dentistry, Osaka University, 1-8 Yamadaoka, Suita 565-0871, Osaka, Japan; sena.usta92@gmail.com (S.Z.U.); ybsk121@yahoo.co.jp (S.K.); kurioka.kyoko.dent@osaka-u.ac.jp (K.K.); dents60032@yahoo.co.jp (T.S.); sugauchi.akinari.dent@osaka-u.ac.jp (A.S.); seki.soju.dent@osaka-u.ac.jp (S.S.); tanaka.susumu.dent@osaka-u.ac.jp (S.T.); 2 Department of Orthodontics & Dentofacial Orthopedics, Graduate School of Dentistry, Osaka University, Suita 565-0871, Osaka, Japan; inubushi.toshihiro.dent@osaka-u.ac.jp; 3 Unit of Dentistry, Osaka University Hospital, 2-15, Yamadaoka, Suita 565-0871, Osaka, Japan

**Keywords:** immune checkpoint inhibitor, resistance mechanism, melanoma, oral mucosal melanoma, immunotherapy, immune checkpoint blockade, anti-PD-1, anti-CTLA-4

## Abstract

Immune checkpoint inhibitors (ICIs), including anti-cytotoxic T-lymphocyte-associated protein 4 (CTLA-4) and anti-programmed death-1 (PD-1) antibodies, have initiated a new era in the treatment of malignant melanoma. ICIs can be used in various settings, including first-line, adjuvant, and neo-adjuvant therapy. In the scope of this review, we examined clinical studies utilizing ICIs in the context of treating oral mucosal melanoma, a rare disease, albeit with an extremely poor prognosis, with a specific focus on unraveling the intricate web of resistance mechanisms. The absence of a comprehensive review focusing on ICIs in oral mucosal melanoma is notable. Therefore, this review seeks to address this deficiency by offering a novel and thorough analysis of the current status, potential resistance mechanisms, and future prospects of applying ICIs specifically to oral malignant melanoma. Clarifying and thoroughly understanding these mechanisms will facilitate the advancement of effective therapeutic approaches and enhance the prospects for patients suffering from oral mucosal melanoma.

## 1. Introduction

Mucosal melanomas are a subtype of melanoma that can develop in various mucosal regions, with the most common locations being the head and neck (55%), anorectum (24%), and vulvovaginal region (18%); are less frequently observed in regions such as the urinary tract; tracheobronchial tree; esophagus; stomach; small and large intestines; gall bladder; and cervix; and oral melanomas constitute 25–40% of head and neck melanomas [1]. Oral melanoma is an uncommon cancer characterized by aggressive progression. It accounts for only 0.2–8% of all melanomas and 1–2% of all oral carcinomas [2]. The cause of oral melanoma and the risk factors contributing to the cells’ malignant transformation remain unclear, and the relationships among human papillomavirus (HPV), human immunodeficiency virus (HIV), and oral mucosal melanoma (OMM) have not been confirmed [3]. The chronic irritations caused by dental prostheses, tobacco use, and infection have been proposed as potential contributors to the onset of oral mucosal melanoma [4].

Oral melanoma holds significant clinical relevance because it has been correlated with a higher mortality rate than cutaneous melanoma. This was because it is often not diagnosed or misdiagnosed in the early stages, and when diagnosed, the disease has often already invaded the surrounding tissues [5,6,7]. Consequently, the complicated anatomical features have limited surgical intervention [5]. This underscores the significance of exploring and implementing effective systemic therapies while encouraging continued research in this field. Furthermore, compared with skin melanoma, fewer treatment options are available for oral melanoma. Therefore, the development of efficient immunotherapy for oral melanoma is vital for improving patient outcomes.

Surgical resection is the standard therapy for treating patients with melanoma [8]. In situations where the lymph node status has the potential to influence treatment planning and the ability to participate in clinical trials, it is advisable to consider a sentinel lymph node (SN) biopsy for accessible sinonasal or oral mucosal melanomas [9]. However, it is not desirable that routine complete lymph node dissection, i.e., completion neck dissection, should be performed on patients with SN-positive oral melanoma [9,10].

Radiotherapy received after the surgery reduced the possibility of local recurrence [11]. Although certain guidelines have suggested photon-based, intensity-modulated radiotherapy after surgical operations in patients with head and neck mucosal melanoma, its effectiveness in addressing distant metastasis has been limited [9,11]. Thus, achieving systemic disease control is of paramount importance, particularly for patients with a heightened likelihood of metastasis.

Dacarbazine has held a notable place in cancer treatment, serving as a commonly used initial chemotherapy option for the management of metastatic melanoma. It exhibited an overall response rate of 13.4%, and the median survival duration varied from 5.6 to 11 months [12]. Nonetheless, due to its limited efficacy, researchers have been driven to investigate more effective treatment modalities, particularly for cases with unresectable and metastatic melanoma, where surgery is not a viable option. For many years, dacarbazine was the standard of therapy, but since 2011, the Food and Drug Administration (FDA)-approved use of immune checkpoint inhibitors (ICIs) and small-molecule inhibitors has brought about substantial changes to the standard treatment approach [13].

The discovery of the *BRAF*^V600E^ mutation was a milestone in the development of targeted and more personalized approaches to melanoma [14]. As a first-line treatment option, targeted therapies such as BRAF and MEK inhibitors have been highly efficient, especially in combination [15].

Despite the great potential of targeted therapies for melanoma treatment, they have some limitations, as only patients with targetable gene mutations are suitable candidates for therapy. Moreover, melanoma treatment that targets a single mutation tends to result in resistance [16,17]. Therefore, additional therapy is required.

Remodeling the immune system to leverage the host’s immune defenses against cancer cells has been an appealing concept for years, and the curated knowledge of the immune system has created opportunities for the development of various immunotherapies.

Interleukin-2 (IL-2) administration represented the first effective immunotherapy for patients with melanoma [18]. A high dose of IL-2 induced a durable anti-tumor response, especially in advanced renal cell carcinoma and melanoma, but severe toxicity was the main obstacle to successful treatment [19]. Furthermore, immunotherapy did not meet expectations until the breakthrough discovery of the immune checkpoint blockade. The groundbreaking research conducted by Honjo et al. in 1992 has led to the discovery of programmed death-1 (PD-1) and its role in immune regulation [20]. PD-1 is predominantly found on T-cell surfaces, whereas programmed death ligand-1 (PD-L1) is present in many cell types, including cancer cells [21]. When PD-1 on T cells binds to PD-L1 on cancer cells, it can lead to the suppression of the immune response, enabling cancer cells to avoid recognition and elimination by the immune system [21]. Thus, blocking PD-1 or PD-L1 has taken center stage in the realm of immunotherapy [22]. Targeting PD-1 and PD-L1 showed good anti-tumor activity, along with less toxicity, than IL-2 therapy [23]. A PD-1 blockade also had a long-term therapeutic effect, compared with conventional chemotherapy that targeted cancer cells, as it targeted T cells instead of cancer cells, which are a heterogenous population [24].

Cytotoxic T-lymphocyte-associated protein 4 (CTLA-4) was another immune checkpoint protein first discovered by Brunet et al. in 1987 [25]. To ensure an efficient and well-regulated immune response, complete T-cell activation necessitates both TCR engagement and the presence of co-stimulatory signals. Among these signals, CD28-mediated co-stimulation, in which the CD28 on T cells binds to CD80/CD86 ligands on antigen-presenting cells (APCs), is important. However, CTLA-4 on T cells engages in competition with CD28 to bind to CD80/CD86, resulting in the inhibition of T-cell activation. Therefore, blocking CTLA-4 activation has emerged as an innovative approach within the field of immunotherapy [26]. After several clinical trials proved the effectiveness of ipilimumab, a monoclonal antibody that targets CTLA-4, the FDA authorized the inclusion of this antibody in the treatment of patients with unresectable or metastatic melanoma [27,28,29].

Currently, ICIs and small-molecule inhibitors play significant roles in melanoma treatment. The American Society of Clinical Oncology (ASCO) guidelines have recommended nivolumab and pembrolizumab as adjuvant systemic therapies for patients with resected stage-IIIA/B/C/D cutaneous melanoma harboring wild-type *BRAF*. For *BRAF*-mutant (V600E/K) patients, in addition to these options, dabrafenib-plus-trametinib has also been recommended. Based on the Checkmate-238 trial, nivolumab has been suggested as an adjuvant therapy for patients with resected stage-IV melanoma.

For patients with unresectable or metastatic melanoma harboring wild-type *BRAF*, ipilimumab-plus-nivolumab, followed by nivolumab, pembrolizumab, or nivolumab, have been recommended. For patients with *BRAF* mutations (V600), in addition to these options, a combination of BRAF/MEK-inhibitor therapy has been recommended. If the disease continues to progress after first-line anti-PD-1 therapy, ipilimumab-containing regimens or therapeutic approaches that combine BRAF and MEK inhibitors have been recommended based on the mutation status. Although primarily intended for patients with cutaneous melanoma, the guidelines stated that these treatment regimens could also be applied to unresectable or metastatic mucosal melanoma [30]. Despite its reduced effectiveness in mucosal melanomas, as compared to cutaneous melanomas, immune checkpoint blockade remains a beneficial treatment option [31].

The United Kingdom’s National Guidelines for Head and Neck Melanoma recommended anti-PD-1 and anti-CTLA4 combination therapy for advanced and metastatic melanoma. If the patient was unsuitable for combination treatment, either nivolumab or pembrolizumab monotherapy was suggested. Depending on the mutation status, either BRAF or c-KIT inhibitors were recommended. If immunotherapy or targeted therapy are not viable choices or resistance occurs, then chemotherapy may be a suitable alternative according to these guidelines [9].

An analysis that examined 52 studies revealed that in head and neck mucosal melanoma, the immunotherapy group consistently demonstrated higher survival rates than the non-immunotherapy group, at 2 years (58% vs. 50%), 3 years (70.1% vs. 42.35%), and 5 years (40.03% vs. 31.7%) [32]. Despite being a groundbreaking treatment approach in melanoma therapy, immunotherapy, especially ICIs, have exhibited significant limitations, including immune-related adverse events (irAEs) and, most importantly, therapy resistance, which continues to be a critical barrier to achieving successful outcomes. However, enhancing our comprehension of resistance mechanisms makes it possible to design treatments that optimize the benefits of ICIs. In the landscape of immunotherapy research, numerous studies have explored the applications of ICIs across various malignancies. However, a notable gap exists when assessing their efficacy and potential in the context of oral malignant melanoma (OMM). To the best of our knowledge, the current literature also lacks a comprehensive review of the status of ICIs in OMM. Therefore, this review gives a comprehensive overview of the current status of ICIs in the treatment of oral mucosal melanoma, as well as the molecular mechanisms of resistance to ICIs, which have been the major obstacle to their effectiveness in melanoma treatment.

## 2. ICI Therapy for OMM

In 1980, Umeda and Shimada proposed a successful treatment regimen for stage-1 and -2 oral melanomas. This protocol involved: (1) performing intraoral surgery to excise the primary lesion; (2) therapeutic radical neck dissection in cases with neck lymph node metastases; and (3) DAV and OK-432 as adjuvant therapy [33]. This approach, incorporating surgical treatment and dacarbazine-based chemotherapy, was considered the standard therapy for patients with stage-1 or -2 oral melanoma.

Another adjuvant therapy is high-dose interferon-α2b (HDI). In clinical trials, the HDI treatment group showed a prolonged relapse-free survival rate (RFS) of 40 months, while the control group had an RFS of 22 months (*p* = 0.0169). However, a significant difference was not detected in terms of overall survival (OS) (i.e., 72 vs. 64 months, respectively, *p* = 0.4236) and RFS (i.e., 53 vs. 34 months, respectively, *p* = 0.1960) between patients diagnosed with stage-III oral mucosal melanoma who were treated with chemotherapy and those who received HDI after chemotherapy as adjuvant therapy. Nevertheless, it has been suggested that for patients with OMM, especially those in stage IVa who do not respond to chemotherapy, HDI can serve as an effective adjuvant therapy, as it extends OS with a notable difference in survival time (40 months compared to 20 months, *p* = 0.0146) [34]. However, the expert panel of ASCO suggested that the advantages of HDI were mitigated by the toxicity, and their impact has been relatively constrained when compared with the effects of more recent, accessible agents. Thus, the routine use of HDI was not within the standard adjuvant therapy recommendations, according to ASCO guidelines [30,35].

Clinical trials have been carried out to evaluate the effectiveness of specific small-molecule inhibitors in an adjuvant setting for melanoma. Patients with completely resected stage-III cutaneous melanoma harboring *BRAF* V600E or V600K mutations showed improved RFS and OS when they received dabrafenib-plus-trametinib as an adjuvant treatment. The combined therapy group exhibited RFS rates of 88% at 1 year, 67% at 2 years, and 58% at 3 years, as compared to 56%, 44%, and 39%, respectively, in the placebo group (Hazard ratio (HR) for relapse, 0.47; 95% CI, 0.39–0.58; *p* < 0.001). Similarly, the estimated OS rates in the combined therapy group were 97% at 1 year, 91% at 2 years, and 86% at 3 years, as compared to 94%, 83%, and 77%, correspondingly in the placebo group (HR for death, 0.57; 95% CI, 0.42 to 0.79; *p* = 0.0006). Nevertheless, the observed difference in the OS rates between the groups was not deemed significant, despite the low *p*-value, as it failed to surpass the predetermined conservative interim boundary of *p* = 0.000019 in the first interim analysis of OS. [36]. Following the outcomes of the COMBI-AD clinical trial (NCT01682083), dabrafenib-plus-trametinib received FDA approval in 2018 as an adjuvant treatment for melanoma patients harboring *BRAF* V600E or V600K mutations [37]. However, in a previous study, although 50–60% of patients with cutaneous melanoma have a *BRAF* mutation, this rate was found to be only 3.5% among 57 patients with OMM [38].

The favorable outcomes achieved with anti-PD-1 and PD-L1 treatment in patients with metastatic and unresectable melanoma have led to the consideration of their potential use as adjuvant therapies. Pembrolizumab was approved by the FDA in 2019 for use as an adjuvant treatment for stage-IIIA (>1 mm lymph node metastasis), -IIIB, or -IIIC cutaneous melanoma after surgery based on the KEYNOTE-054 pivotal trial, which had demonstrated that the pembrolizumab group had significantly prolonged, recurrence-free survival, as compared to the placebo group (with a HR for recurrence or death of 0.57; 98.4% CI, 0.43 to 0.74; *p* < 0.001) [39,40]. Furthermore, in 2021, it received FDA approval as an adjuvant treatment for patients aged 12 years and older with stage-IIB or -IIC melanoma following a complete resection, based on the KEYNOTE-716 (NCT03553836) trial, which had demonstrated a significant improvement in RFS for the pembrolizumab treatment group, as compared to the placebo group during the first interim analysis with a HR of 0.65 (HR 0.65; 95% CI 0.46–0.92; *p* = 0.0132) [41]. Adjuvant pembrolizumab was also recommended for resected stage-IIB or -IIC melanoma in the ASCO guidelines, based on the findings of the KEYNOTE-716 trial. Adjuvant nivolumab was recommended in ASCO guidelines for resected stage-IIB or -IIC melanoma due to the similar outcomes and toxicity profiles between the CheckMate 76K (HR 0.42; 95% CI 0.30–0.59; stratified *p* < 0.0001) and the KEYNOTE-716 trial [30,42]. However, the data on OS for adjuvant pembrolizumab and nivolumab were unavailable. Additionally, no positive trials for stage-IIA disease existed, leading to their exclusion from the recommendation for stage-IIA melanoma [30].

Studies have shown that patients with nodular-type oral mucosal melanoma who were treated with chemotherapy—dacarbazine and cisplatin—plus anti-PD-1 agents as adjuvant therapy showed improved 2-year OS (71.0%; *p* (Chemotherapy vs. Chemotherapy + Anti-PD-1)  0.0118) and progression-free survival (PFS) (53.6%; *p* (Chemotherapy vs. Chemotherapy + Anti-PD-1)  0.0001), along with less cytotoxic effects (16% vs. 59%, (*p*  <  0.0001), while decreasing the likelihood of melanoma recurrence in the oral (Chemotherapy + IFN vs. Chemotherapy + Anti-PD-1, *p* = 0.71) and distant regions (Chemotherapy + IFN vs. Chemotherapy + Anti-PD-1, *p* = 0.047), as compared to patients who received chemotherapy plus high-dose interferon-α2b (HDI) [43].

In a double-blind, phase-III trial (EORTC 18071) involving patients with stage-III cutaneous melanoma, researchers found that after complete resection, an adjuvant treatment with intravenous infusions of ipilimumab (CTLA-4 inhibitor) at a dosage of 10 mg/kg, initially given every 3 weeks for 4 doses, followed by its administration every 3 months for a maximum duration of 3 years, led to a noteworthy improvement in RFS, as compared to the control group (*p* = 0.0013). In light of the findings from this study, the FDA authorized the use of ipilimumab as an adjuvant treatment for high-risk stage-III melanoma after complete resection. A notable percentage of patients in the treatment group (245 out of 471) experienced side effects leading to treatment discontinuation, while in the control group, 20 out of 474 patients experienced similar outcomes [44,45]. Due to the high cost and severe toxicity associated with the use of ipilimumab as an adjuvant therapy, its use was not recommended in adjuvant settings [46,47].

In a clinical trial comparing adjuvants nivolumab and ipilimumab in patients with resected stage-III or -IV melanoma, the 18-month RFS in patients treated with nivolumab was 66.4%, whereas in patients treated with ipilimumab alone, it was 52.7%. Additionally, the ipilimumab-treatment group demonstrated a drug-related death rate of 0.4%, whereas there were no recorded fatalities attributed to drug-related issues in the nivolumab-alone group. Moreover, drug-related grade-3 (severe or medically significant) and grade-4 (life-threatening) side effects, including fatigue, diarrhea, pruritus, rash, nausea, arthralgia, asthenia, hypothyroidism, headache, abdominal pain, increased ALT levels, increased AST levels, maculopapular rash, hypophysitis, and pyrexia, were observed in 45.9% of patients in the ipilimumab group, whereas only 14.4% of the patients in the nivolumab group experienced these side effects. These results indicated that nivolumab was safer than ipilimumab [48]. However, the scarcity of mucosal melanoma cases, particularly OMM, resulted in a restricted number of clinical trials exploring the utilization of ICIs in adjuvant therapy.

When surgery is not indicated, such as in patients with unresectable or metastatic melanoma, targeted therapy and immunotherapy have emerged as the preferred initial treatment options because the efficacy of chemotherapy in terms of OS has been notably limited [49]. BRAF inhibitors, particularly in combination with MEK inhibitors, have proven to be highly efficient in patients with cutaneous melanoma; however, these mutations have seldom been found in mucosal melanoma [50]. In cases where a targetable mutation exists in the patient, targeted therapy may be a potential candidate as a first-line treatment.

A patient with OMM harboring a KIT mutation underwent targeted therapy for this mutation with a KIT inhibitor, and no signs of recurrence were detected within 41 months [51]. Moreover, in patients with metastatic OMM with a KIT mutation, it was reported that treatment with imatinib extended OS, as compared to conventional chemotherapy. However, 5 out of 12 patients died due to treatment resistance [16]. Thus, the inherent heterogeneity of melanoma necessitated a multifaceted approach, targeting not only the individual mutations but also various mutation patterns or pathways in combination, to enhance the response rate to treatment.

ICIs are of paramount importance in melanoma treatment, especially when patients lack a targetable mutation. In a pooled data analysis, patients who received nivolumab alone experienced a median PFS of 3.0 months for mucosal melanoma and 6.2 months for cutaneous melanoma, with objective response rates (ORRs) of 23.3% and 40.9%, respectively. However, the most significant outcome was observed in patients who received nivolumab and ipilimumab in combination, where the median PFS increased to 5.9 months for mucosal melanoma and 11.7 months for cutaneous melanoma, with increased ORRs of 37.1% and 60.4%, respectively [52]. This suggested that combination therapy had more significant and favorable outcomes in terms of response rates and PFS in patients with either type of melanoma. However, the patients undergoing combination therapy exhibited a notably higher occurrence of side effects, as evidenced by the data. Among those with mucosal melanoma who received nivolumab monotherapy, any-grade side effects occurred in 66.3% of cases, with grade-3 and -4 side effects in 8.1%, while in the case of combination therapy for mucosal melanoma, any-grade side effects were observed in a striking 97.1% of instances, with grade-3 and -4 side effects occurring in 40% of the cases [52]. While toxicity management may vary slightly depending on the affected system or region, for most organ categories and generally through dose delay and/or adverse event management with steroids (immunosuppressive agents, immunomodulatory agents, secondary immunosuppressive agents), the resolution of grade-3–4 adverse side effects was observed in 67–100% of cases, except for endocrine-related side effects in the combination therapy group. Endocrine-related side effects were managed with hormone replacement therapy; however, only one-quarter of them were resolved [53].

A post hoc analysis of the KEYNOTE-001, 002, and 006 studies evaluated the effectiveness of pembrolizumab in advanced mucosal melanoma cases; the overall ORR in patients with mucosal melanoma was 19%, with a median PFS of 2.8 months and a median OS of 11.3 months. Notably, the responses were comparable between ipilimumab-naïve and ipilimumab-treated patients, indicating that pembrolizumab showed promising efficacy regardless of previous ipilimumab exposure [54]. In conclusion, PD-1 inhibitors have been effective in the treatment of melanoma, both as a monotherapy and in combination therapy, offering promising results for patients with different melanoma subtypes.

Oral amelanotic melanoma, a subtype of oral melanoma without pigmentation, constitutes 75% of oral melanoma cases and presents diagnostic challenges due to the absence of typical melanin pigmentation, potentially resulting in a poorer prognosis, as compared to pigmented melanomas [55,56]. In a patient with metastatic oral amelanotic melanoma stage IVc with negative PD-1 levels, it was observed that combination therapy with ipilimumab at 3 mg/kg and nivolumab at 1 mg/kg administered every three weeks for four cycles visibly decreased the size of the oral melanoma lesion, along with the shrinkage of the metastatic lesions. However, following the administration of the second immunotherapy dose, the patient suffered from severe adverse side effects, such as myocarditis, hypophysitis, and neuritis. After stopping treatment with the combined immune checkpoint blockade, the patient was treated with high-dose steroids (1000 mg of solumedrol per day, divided into 4 doses). Upon achieving near-normal CPK levels, improved breathing, and a normalized heart rate, the steroid doses were gradually tapered off, with prednisone starting at 2 mg/kg and then reducing the dose by 7.5% daily. At a one-month follow-up, the patient had a regular heart rate, a normal rhythm, and resolved hypophysitis, as evidenced in imaging and laboratory results, with no evidence of cranial nerve neuritis. The patient was successfully weaned off steroids, but unfortunately, sudden cardiac death occurred shortly thereafter [57].

In a patient with extensive advanced oral melanoma, treatment with ipilimumab followed by pembrolizumab showed a favorable response. This response was so effective that it eliminated the need for surgery in cases where there had been no tumor progression or recurrence [58]. However, to observe the long-term, reliable effects of pembrolizumab on mucosal melanoma, larger patient cohorts and longer-duration clinical studies are needed.

In addition to being used as a first-line and adjuvant treatments, ICIs have enhanced the effects of other conventional therapies, such as radiation and chemotherapy, according to recent research findings.

The term “abscopal effect” refers to a rare phenomenon in which radiation therapy applied to one area exerts an anti-tumor effect on a distant tumor. This effect was observed in a patient with OMM who underwent a maxillary resection and a bilateral neck dissection, followed by an adjuvant treatment with nivolumab. After the occurrence of brain, spleen, and liver metastases, the patient received radiation therapy for the brain tumor, and as a result of the abscopal effect, a regression was seen in the liver and spleen metastases. Nivolumab may have played a role in exerting this effect [59].

ICIs have also improved the effectiveness of subsequent chemotherapy. In patients with malignant melanoma who developed resistance to PD-1 blockades, the overall response to chemotherapy administered with a PD-1 inhibitor was higher than in patients who received chemotherapy alone [60]. Recent studies have investigated the use of ICIs in conversion therapy. Conversion therapy involves shrinking initially unresectable cancerous lesions through various treatment approaches, such as chemotherapy, immunotherapy, and radiotherapy, to make surgical operations feasible and safe. Zhang et al. demonstrated the effectiveness of combining PD-1 inhibitors with tyrosine kinase inhibitors as a conversion treatment for advanced hepatocellular carcinoma, showing an RFS rate of 75% at 12 months after surgery [61]. However, clinical studies on the use of ICIs as conversion therapy for melanoma and other types of cancer have been limited. Especially in OMM, where a late diagnosis can make surgery extremely challenging, the application of ICIs as conversion therapy is promising and merits further research.

Numerous clinical trials have been conducted and are planned for advanced melanoma (Table 1). However, there are fewer clinical trials for mucosal melanoma due to its rarity, as compared to cutaneous melanoma. Particularly, patients with oral mucosal melanoma should be encouraged to participate in clinical trials, as this is crucial due to the condition’s rarity and limited treatment options; the increasing potential for personalized therapies and advancements; and the contribution to scientific progress.

## 3. Immunotherapy Resistance

Resistance to immunotherapy can be clinically classified into two main categories. Patients who do not experience any therapeutic benefits from the initial attempt are considered to have primary resistance to the therapy. In contrast, some patients may initially benefit from the therapy, but over time, the tumor cells can acquire resistance, known as an acquired resistance through adaptive changes, leading to tumor regrowth [62]. This resistance can be triggered intrinsically or extrinsically.

The discussion of resistance mechanisms primarily relies on cutaneous melanoma data, as these provide well-studied and more prevalent models in the field. The data offer insights into the broader understanding of melanoma resistance, even though this article’s main focus is on oral mucosal melanoma. However, it is crucial to recognize the unique characteristics of mucosal melanoma and its potential differences in resistance mechanisms in order to advance treatments for this less common subtype.

### 3.1. Mechanism of Intrinsic Resistance to Immune-Checkpoint-Blockade Therapy in Melanoma

Tumor-cell-intrinsic resistance mechanisms to immunotherapy refer to the specific genetic and molecular alterations within tumor cells that hinder immune cell infiltration and function, leading to resistance against immunotherapy [62].

**Figure 1:**
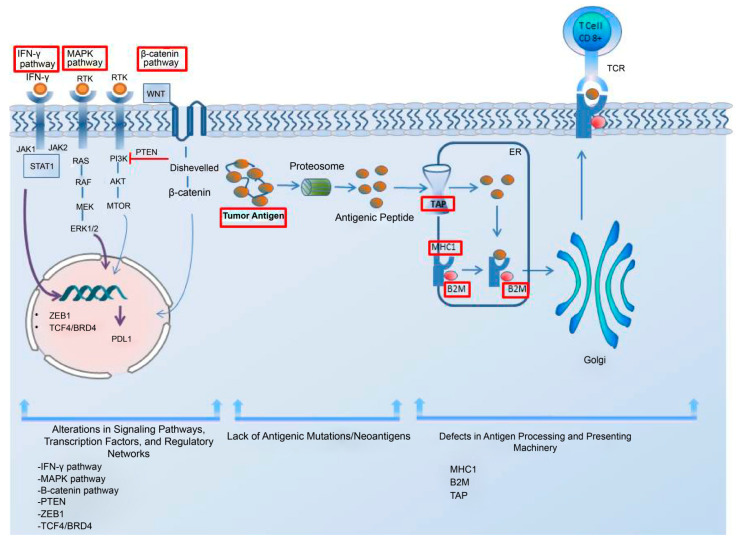
The intrinsic mechanism underlying the resistance to immune checkpoint blockade in melanoma cells. This intrinsic mechanism includes the following elements: 1. alterations in signaling pathways, transcription factors, and regulatory networks; 2. the lack of antigenic mutations/neoantigens; and 3. defects in the antigen processing and presenting systems. The upregulation of the MAPK pathway can contribute to resistance by increasing the PDL-1 expression, impairing T-cell infiltration and, inducing the acquisition of a mesenchymal phenotype by melanoma cells. The activation of the β-catenin pathway leads to T-cell exclusion and resistance, as does the loss of PTEN and the upregulation of the transcription factor ZEB1. The lack of tumor antigens and antigenic mutations poses a barrier to the immune system’s ability to recognize tumor cells. A regulatory network dependent on TCF4/BRD4 not only hinders antigen presentation, interferon signaling, and the activation of leukocyte-associated gene expression but also supports the acquisition of a mesenchymal phenotype, leading to resistance. For an effective immune response, tumor antigens need to be presented to the immune system. The dysregulation of MHC-1, B2M, and TAP in the antigen processing and presenting machinery hinders proper antigen presentation, thereby impeding a robust immune response. Interferon-gamma plays a dual role in resistance mechanisms by upregulating both MHC-1 and MHC-2, contributing to antigen processing and presenting machinery, while also leading to PD-L1 expression. Abbreviations: MHC, major histocompatibility complex; TAP, the transporter associated with antigen processing; B2M, beta-2-microglobulin; IFN-γ, interferon-gamma; MAPK, mitogen-activated protein kinases; PTEN, phosphatase and tensin homolog; ZEB1, zinc finger E-box-binding homeobox 1; ER, endoplasmic reticulum.

Low antigen expression is one of the primary contributors to the resistance to immunotherapy in cancer cells. Additionally, the impairments in the system responsible for antigen processing and presentation; the upregulation of constitutive PD-L1; the absence of tumor-specific antigens and antigenic mutations; perturbations in the signaling pathways; the genetic exclusion of T cells; and the modifications in immune evasion mechanisms may significantly contribute to the evasion of immune responses, as elucidated by Sharma et al. in their comprehensive review [62]. In addition, the regulatory networks dependent on TCF4/BRD4, MYC, the cytoprotective enzyme heme oxygenase-1 (HO-1), the loss of Kelch-like ECH-associated protein 1 (KEAP1), and the loss of E-cadherin have been considered resistance-driver factors against immune checkpoint blockades in melanoma patients [63,64,65,66,67]. The intrinsic mechanism underlying the resistance to immune checkpoint blockade in melanoma cells is illustrated in Figure 1.

#### 3.1.1. Impairments in the Antigen Processing–Presenting Machinery

Therapies targeting CTLA-4 and PD-L1 promote T-cell-driven immune enhancement against cancer. For these therapies to be effective, T cells present in the environment must recognize the cancer cells. Antigen presentation stimulates T cells to recognize the pathological cells; thus, any defect in the components of the tumor-antigen-presenting machinery that prevents T cells from recognizing cancer cells may facilitate the evasion of immune defenses by tumor cells.

Human leukocyte antigen class I (HLA-I) is a noteworthy component of this machinery. Analysis of transcriptomic data from melanoma biopsies of immune checkpoint blockade responders and non-responders showed that responders had high levels of HLA-I in comparison to non-responders, suggesting that the suppression of the HLA-I antigen processing-and-presentation machinery played a significant role in the primary resistance to anti-CTLA-4 and anti-PD-1 therapy. Alternatively, the induction of retinoic acid-inducible gene I (RIG-I) was demonstrated to reverse HLA-I suppression in patients with melanoma, suggesting that it could be targeted to overcome resistance to immune checkpoint blockades [68]. Of note, Paulson et al. emphasized the importance of distinguishing between the two types of immunotherapy escape mechanisms, genetic HLA loss and transcriptional HLA loss, as genetic HLA loss demands the generation of novel T-cell responses to target alternate HLAs in order to overcome immunotherapy resistance, while transcriptional HLA loss had the potential to be reversed through drug-based therapies in order to restore HLA expression [69]. This study underscored the importance of recognizing these distinct mechanisms to better understand and develop effective strategies for overcoming immunotherapy resistance.

Beta-2-microglobulin (B2M) is another indispensable element of antigen processing–presenting machinery (APM) that participates in the MHC class-I antigen presentation; its loss, particularly through the loss of heterozygosity, may lead to the subsequent loss of MHC Class 1 and the proper presentation of tumor antigens, which then hampers an effective anti-tumor response and contributes to immune evasion and resistance to therapy. The presence of B2M defects in patients was significantly correlated to non-responsiveness to anti-CTLA-4 therapy and anti-PD-L1 therapy. Notably, the fact that B2M defects were predominantly detected in samples taken before treatment from non-responders, as well as in post-progression samples from patients with an initial response to immune checkpoint blockade, suggested that B2M alterations may be involved in both acquired and primary resistance in metastatic melanoma [70].

IFN-γ displayed a critical role in the antigen processing-and-presentation machinery, predominantly by upregulating MHC-1 and MHC-2 [71]. This became significant, especially within the scope of anti-PD-1 therapy, given that the initial response to such treatment was linked to preexisting immune activation mediated by IFN-γ, which involves the expression of MHC-2 in metastatic melanoma [72]. IFN-γ also had a role in inducing PD-L1 expression [73].

Studies have indicated that impairments in the IFN-γ signal pathway in melanoma could also lead to a reduced response to anti-CTLA-4 therapy [74]. In contrast, prolonged IFN-γ exposure caused resistance to radiotherapy and anti-CTLA-4 treatment in melanoma cells. This was because IFN-γ increased the PD-L1 expression, which suppressed the T cells and resulted in adaptive resistance. Furthermore, extended IFN-γ exposure induced adaptive resistance through the STAT1 pathway, independent of its impact on PD-L1 expression [75]. This indicated that IFN-γ has a multifaceted role in inducing resistance to immune-checkpoint-blockade therapy, contributing to primary, adaptive, and acquired resistance in melanoma.

#### 3.1.2. Alterations in Signaling Pathways

One of the resistance driver pathways is the MAPK pathway, whose upregulation negatively affected anti-tumor activity by regulating the production of VEGF and IL-8. The upregulation of these cytokines resulted in defective T-cell infiltration [62]. Jiang et al. showed that the MAPK pathway had the potential to stimulate PD-L1 expression in melanoma cells that had developed resistance to BRAF inhibition, suggesting that targeting the MAPK pathway could enhance the tumor response to immunotherapy [76]. Furthermore, mutations in this pathway increased CD73 expression. It has been suggested that these enzymes were upregulated in some patients undergoing immune-checkpoint-blockade therapy, and the melanoma cells acquired a mesenchymal phenotype, ultimately causing adaptive resistance to therapy [77].

T-cell exclusion is another critical factor that has led to resistance to ICI therapy. It was observed that the activation of the β-catenin pathway in melanoma cells led to T-cell exclusion and the primary resistance to T-cell-based cancer treatments, such as ICIs, due to the insufficient number of pre-existing T cells [78]. Tumor-intrinsic β-catenin signaling caused the defective recruitment of CD103^+^ dendritic cells, which are crucial for CD8^+^ T-cell function and immune infiltration [78].

PTEN is another determinant of T-cell exclusion. Zhao et al. examined 66 patients with glioblastoma multiforme, before and after PD-1 therapy, to investigate the determinants of the therapeutic response. They detected an enrichment of PTEN mutations in non-responders, indicating that PTEN loss was connected to the development of resistance to anti-PD-1 therapy [79].

The loss of PTEN contributed to resistance to immunotherapy in melanoma, mainly by activating the PI3K pathway and decreasing the level of CD8 T cells in tumors through the secretion of immunosuppresive cytokines as well as by inhibiting autophagy [80].

In contrast, ZEB1, a transcription factor that promotes the transition from epithelial to mesenchymal states, may also contribute to immune escape by decreasing CD8^+^ T-cell accumulation in melanoma. ZEB1 caused a decrease in CD8^+^ T-cell infiltration into tumors through the downregulation of CD8^+^ T-cell-attracting chemokines and cytokines, such as CXCL10, CCL3, CCL4, IFN-γ, and TNF-α. Thus, ZEB-1 depletion was a positive regulator of anti-PD1 therapy [81].

#### 3.1.3. Absence of Tumor Antigens and Lack of Antigenic Mutation

Tumor neoantigens produced by tumor cells are distinct antigens formed by specific genetic mutations in cancerous cells. These mutations make them recognizable as foreign cells by the immune system and initiate an immune reaction against the tumors. However, tumor cells may sometimes lack these tumor antigens or may have some limitations in presenting them on the cell surface. This leads to an ineffective T-cell response and, in turn, resistance to T-cell-based immunotherapies, such as anti-PD-L1 and anti-CTLA-4, in melanoma.

However, the chronic stimulation with tumor antigens also promoted T-cell dysfunction and unresponsiveness [82]. Thus, antigenic mutations and newly emerged neoantigens were required to boost an effective anti-tumor response. Newly emerged neoantigens can serve as crucial factors in inducing durable T-cell responses and overcoming resistance. Studies have shown that the synergistic action of newly emerged neoantigen-induced CD8^+^ T cells and anti-PD-L1 therapy contributed to tumor elimination in a murine model of malignant melanoma [82]. The isolation of neoantigen-specific TCRs from metastatic melanoma tumor samples indicated the existence of neoantigen-specific T-cell responses [83]. Tumors with a high abundance of clonal neoantigens were more responsive to immune checkpoint blockades in patients with melanoma [84]. Nevertheless, it is important to highlight that even melanomas with a low abundance of neoantigens have exhibited positive responses to immune checkpoint therapy [85]. Thus, understanding the role of clonal neoantigens in immune-checkpoint-blockade resistance is crucial for developing strategies to overcome resistance and improve treatment outcomes. Efforts are underway to identify and characterize the neoantigens in melanoma and to develop personalized immunotherapies that target these specific antigens, potentially enhancing the effectiveness of immune checkpoint blockades in resistant tumors.

#### 3.1.4. Expression of PD-L1 and Other Contributing Factors to Resistance

The relationship between PD-L1 expression and resistance to immune checkpoint therapy is complex and needs to be clearly understood to select suitable therapies for patients and improve their prognosis.

PD-L1 can be either constitutively expressed or induced. The constitutive expression of PD-L1 was triggered by intrinsic oncogenic signaling pathways, whereas inducible PD-L1 was expressed as a response to inflammatory cytokines in the tumor microenvironment (TME) [86].

Intrinsic PD-L1 had a pro-tumoral effect, and this led to responsiveness to PD-1/PD-L1-inhibitor therapy [87]. Recent findings have indicated that melanoma cells displaying increased constitutive PDL-1 expression had a diverse transcriptomic profile characterized by de-differentiation and active tumor necrosis factor (TNF) and interferon (IFN) signaling pathways, potentially contributing to resistance [88].

The presence of enhancers in patients before treatment or their acquisition during treatment could also lead to innate or adaptive resistance to ICI therapy in melanoma by activating several pathways that caused resistance [89].

In a recent study, a regulatory network dependent on TCF4/BRD4 was linked to the development of resistance to both targeted and immune checkpoint therapies in melanoma. This network supported the maintenance of a mesenchymal-like phenotype while inhibiting gene expression associated with antigen presentation, interferon signaling, and the activation of leukocytes [63]. MYC appeared to have a substantial influence on immunotherapy resistance by negatively affecting Janus Kinase 2 (JAK2) expression and the responsiveness of melanoma cells to IFN-γ [64].

Fructose consumption has also been shown to upregulate the cytoprotective enzyme heme oxygenase-1 (HO-1), which contributed to resistance to checkpoint blockades in melanoma mouse model [65]. This indicated that dietary factors could influence TME and potentially hinder the effectiveness of ICIs.

Furthermore, the loss of KEAP1 in melanoma was identified as a factor leading to resistance against anti-PD-1 therapy. Patients with low KEAP1 expression, when treated with an anti-PD-1 antibody, exhibited worse OS compared with patients with high KEAP1 expression [66]. This suggested that the status of KEAP1 expression may serve as a predictive biomarker for patient responses to anti-PD-1 therapy. Furthermore, eIF4F, a eukaryotic translation initiation complex, has a potential role as a predictive marker, as it was correlated to a positive response to ICI therapy in patients with mucosal melanoma [90].

In addition, resistance to immune checkpoint blockade was connected to the loss of E-cadherin, which was indicative of mesenchymal transition [67]. E-cadherin loss in tumor cells may inhibit CD103 anti-tumor activity and diminish the effectiveness of immune checkpoint blockade [67]. These observations highlighted the intricate interplay of the factors contributing to resistance to immune-checkpoint-blockade therapy in melanoma and emphasize the need for more precise and targeted approaches.

### 3.2. Role of the Extrinsic Tumor Resistance Mechanism

Tumor cell-extrinsic resistance mechanisms in immunotherapy include factors external to events within the tumor cells, such as immune-suppressive cells, inhibitory receptors [62], hypoxia, and phenotype switching that cause resistance to immunotherapy.

**Figure 2:**
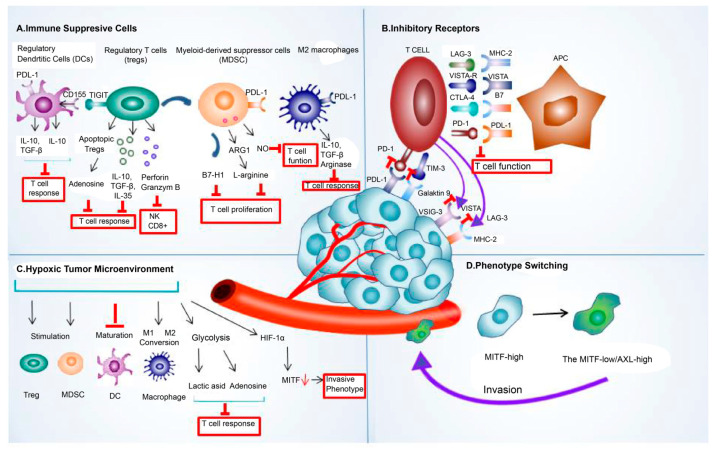
The extrinsic mechanism underlying the resistance to immune checkpoint blockade. This mechanism of resistance to immune checkpoint blockade includes extrinsic (**A**) immune-suppressive cell components, such as regulatory T cells (Treg), tumor-associated macrophages (TAMs), regulatory/tolerogenic dendritic cells (DC), and myeloid-derived suppressor cells (MDSC); (**B**) various inhibitory receptors, such as PDL-1, CTLA-4, LAG-3, TIMS-3, and VISTA; (**C**) hypoxic tumor microenvironment; and (**D**) phenotype switching. Abbreviations: LAG-3, lymphocyte-activation gene 3; PDL-1, programmed death ligand 1; CTLA-4, cytotoxic T-lymphocyte-associated protein 4; TIM-3, T-cell immunoglobulin and mucin domain 3; VISTA, V-domain I g suppressor of T cell activation; HIF-1α, hypoxia-inducible factor 1-alpha; MITF, microphthalmia-associated transcription factor.

The crosstalk between TME components, such as immunosuppressive cells, inhibitory receptors, exosomes, and cancer cells, allowed cancer cells to escape immune attacks by amplifying the immune-suppressing environment [91]. The abstract of the extrinsic mechanism of resistance to immune checkpoint blockade is shown in Figure 2.

Tregs represent an important subgroup of immunosuppressive cells that infiltrate the melanoma microenvironment [92]. Treg cells employ their ability to dampen the immune response by producing cytokines like IL-10 and IL-35 [93]. Aside from their ability to suppress effector T cells, they induced tumor-infiltrating macrophages to generate B7-H molecules, and the interaction of these molecules with their ligands contributed to immune tolerance by dampening the T-cell response [94]. Additionally, Tregs could communicate with other immunosuppressive cells in the TME by secreting various cytokines, which in turn enhanced the immunosuppressive microenvironment [93]. Nevertheless, even after their death, Treg cells could persist in exerting their immunosuppressive effects [95].

A study revealed that Tregs underwent programmed cell death within the TME, and contrary to expectations, apoptotic Tregs were more effective at suppressing T-cell activation, mainly by releasing high levels of ATP and converting it into immunosuppressive adenosine using specific enzymes. It was also shown in mouse cancer models that apoptotic Tregs adversely affected anti-PDL-1 therapy. This study proposed that hypoxia-induced Treg apoptosis could serve as a novel immune evasion mechanism in the TME, potentially leading to immune-checkpoint-blockade resistance [96].

Myeloid-derived suppressor cells (MDSCs) are a diverse group of myeloid cells with immunosuppressive capabilities [93]. One of the most important mechanisms underlying their immunosuppressive activity is the increased expression of nitric oxide (NO) and arginase (Arg)-1. Arg-1 causes L-arginine depletion, which is required for T-cell function, whereas NO expression inhibits T-cell proliferation. Through these mechanisms, MDSCs led to T-cell dysfunction and a reduced response to immunotherapies, such as immune checkpoint blockade [97].

High MDSC levels in melanoma patients who received ipilimumab indicated an unfavorable prognosis [98]. Conversely, patients with advanced melanoma who had reduced levels of CD33^+^CD11b^+^HLA-DR^−^MDSCs before treatment with ipilimumab exhibited extended survival and an objective clinical response [99]. Studies also provided evidence regarding the association between an increased MDSC population and a lack of functional T cells [100], which could limit the efficacy of ICIs. Another study identified endogenous opioids as the potential drivers of T-cell dysfunction and the resistance to immune checkpoint blockade in melanoma [101].

Cell-to-cell interactions also have a notable impact on shaping the TME. Cells in the TME may have the ability to impact the characteristics of cancer cells and the expression of other cells in the TME. Tirosh et al. examined 471 tumors from the Cancer Genome Atlas dataset. They found that a TME in which cancer-associated fibroblasts (CAFs) were highly abundant was associated with an invasive phenotype (microphthalmia-associated transcription factor (MITF)-low/AXL-high) of melanoma cells [102]. Phenotype switching is the term used to describe the ability of melanoma cells to transition between various cell states [103]. Resistance to PD-1 inhibitors, a common type of ICIs, has often been associated with melanoma de-differentiation [104]. An investigation of melanoma tumor tissue samples taken before the administration of anti-PD-1 therapy revealed that patients who had responded to the therapy showed differentiated gene signatures characterized by high proliferation and low invasiveness. In contrast, non-responders were characterized by highly invasive and de-differentiated gene expression signatures [105]. These findings emphasized that by undergoing a phenotypic switch from proliferative, highly differentiated MITF subpopulations to invasive, de-differentiated MITF subpopulations, melanoma cells potentially became resistant to ICB therapy.

IDO, mainly present in tumor and host immune cells, is an enzyme that degraded tryptophan and negatively regulated the immune response to immune checkpoint blockades by inducing T-cell exhaustion and Treg proliferation in the TME [106,107]. IDO-deficient mice showed elevated intratumoral ratios of effector T cells to T regs, which was a positive indicator of prognosis after treatment with CTLA-4 [108]. Its deficiency was also shown to improve the prognosis after anti-PD-1/PDL-1 treatment [108].

Thus, a combination therapy of IDO inhibitors and ICIs has emerged to overcome resistance and enhance the efficacy of the treatment. Although phase-I/II trials of the IDO inhibitor-plus-pembrolizumab in solid tumors, including melanoma, showed promising results, a phase-III trial in patients with unresectable or metastatic melanoma failed due to not meeting the primary end-point [109,110], and no significant difference between the combination treatment and pembrolizumab-alone groups was observed [110].

However, some studies showed that, while elevated IDO expression in surgically treated patients was correlated to shorter PFS or OS, treatment with PD-1 inhibitors showed longer PFS in patients with acral and mucosal melanoma who had elevated IDO levels. Iga et al. stated that the reason for this was that the immune-suppressive environment caused by IDO could be reversed with immunotherapy, but the immune-suppressive environment created by IDO in patients treated with surgery alone could not be reversed [111]. In essence, the conflicting findings suggested that IDO may have a dual role in cancer. In patients treated with surgery alone, IDO’s immune-suppressive effects may worsen outcomes. Nonetheless, in patients receiving immunotherapy, the therapy may counteract these effects, leading to better responses.

TAMs are another cell component present in TME. TAMs exhibit various phenotypes, encompassing M1-like and M2-like characteristics, with the latter being associated with immunosuppressive functions. In an experimental mouse model of melanoma that used a monoclonal antibody against MARCO, a scavenger receptor on TAMs reduced the presence of M2 TAMs and improved the effectiveness of anti-CTLA-4 antibody therapy [112]. Several mechanisms have been proposed to explain how TAMs promote resistance to immune checkpoint therapy. One mechanism is through the secretion of immunosuppressive molecules, such as TGF-β and PGE2. These molecules inhibited the activity of cytotoxic T cells and promoted the expansion of Tregs, which dampened anti-tumor immune responses. Additionally, TAMs could produce immune checkpoint proteins, like PD-L1, which could then directly suppress the activity of T cells. The presence of PD-L1 in TAMs was correlated to a resistance to PD-1/PD-L1 blockade therapy [113].

Additionally, hypoxia is a significant factor in influencing how patients with melanoma respond to ICIs and develop resistance to them. Hypoxia reduced the expression of MITF, a crucial gene involved in melanocyte differentiation. This decrease in MITF levels was controlled by hypoxia-inducible factor 1α (HIF-1α). Consequently, melanoma cells adopted a more invasive phenotype, which has the potential to lead to resistance against immunotherapy [114]. As with chronic antigen stimulation, hypoxia could also affect multiple facets of T-cell function and contribute to therapeutic resistance by causing T-cell dysfunction. Moreover, lactate induced by hypoxia encouraged Tregs to maintain their immunosuppressive properties and regulate the expression of PD-1 and PDL-1 [115,116]. In addition, cells exposed to hypoxia could undergo a hypermetabolic transformation marked by elevated glycolytic activity, leading to resistance [117].

In the case of an excessive immune response, a regulatory mechanism came into effect to avoid the excessive stimulation of T cells that could cause autoimmune diseases and tissue damage. In response, the cells released inhibitory molecules, such as CTLA-4; PD-1/PD-L1; T-cell immunoglobulin and mucin domain 3 (TIM-3); and lymphocyte-activation gene 3(LAG-3), for immune modulation [118]. The effectiveness of ICIs could be hindered in melanoma because of the increased expression of immune checkpoints, such as TIM-3 and V-domain Ig suppressor of T-cell activation (VISTA) [119]. Preclinical studies also correlated TIM-3 upregulation to a resistance to anti-PD-1 therapy, and elevated VISTA expression was observed in patients with melanoma who underwent disease progression while receiving anti-PD-1 inhibitor therapy [119]. The T-cell immunoreceptor with Ig and ITIM domains (TIGITs), a recently identified immune checkpoint receptor, interacted with its ligand CD155, transmitting inhibitory signals and thus functioning like other immune checkpoints, such as PD-1 and CTLA-4 [120]. Therefore, therapies aimed at blocking these co-inhibitory receptors to counteract immune resistance by reversing the negative effects they exert on T cells are in development.

## 4. Future Directions

Combination therapies targeting specific pathways and proteins are at the forefront for overcoming immune-checkpoint-blockade resistance and enhancing the treatment response. Combination therapies using oncolytic viruses and ICIs are among the most promising approaches.

An oncolytic virus refers to a virus modified through genetic engineering or found naturally that specifically targets and reproduces within cancer cells, thus triggering a specific anti-tumor immunity, leading to the death of cancer cells [121]. Oncolytic viruses assisted in overcoming the resistance to immune-checkpoint-blockade therapy by infecting cancer cells and inducing their lysis, resulting in cell death. They also stimulated anti-tumor immunity, converting a cold TME into a hot TME [122,123].

In patients with advanced melanoma, combination therapy with pembrolizumab and talimogene laherparepvec (T-Vec), a double-mutated, second-generation oncolytic herpes simplex virus type 1 (HSV-1), resulted in a notable ORR of 62% and a complete response rate of 33%. In patients who showed treatment efficacy, combination therapy could modify the TME by enhancing various cell populations, primarily that of CD8^+^ cells [124].

When combined with anti-CTLA-4, G47Δ, a triple-mutated oncolytic HSV-1, increased the influx of effector T cells into the TME in various cancer mouse models, including melanoma. This combination therapy augmented the effectiveness of systemic treatment, while also promoting the conversion of initially immune-resistant TME to one that could respond favorably to the immune response [125].

G47Δ was highly effective in mouse models of oral squamous cell carcinoma, preventing cervical lymph node metastasis by viral trafficking from the primary lesion to the lymph node [126]. Especially in cases of oral melanoma where the risk of metastasis is considerably higher due to late diagnosis, the combination of G47Δ with ICIs holds considerable potential.

Targeting TGF-β is promising in overcoming resistance and enhancing responses to immunotherapy. TGF-β had a complex role in tumor immunity, hampering the ability of T cells to infiltrate tumors, reducing their effector functions, and promoting the differentiation of peripheral CD4^+^ T cells into Tregs, potentially limiting the host’s natural defense against cancer [127]. To counteract these effects and boost the efficacy of anti-PD-L1 treatment, researchers have proposed blocking TGF-β [128]. One promising avenue in this endeavor involved the development of Fc fusion proteins that specifically target TGF-β. This fusion protein, TβRI-TβRII-Fc chimeric receptor, could counteract TGF-β-induced mesenchymal properties in malignant melanoma cells in vitro and reduce B16 melanoma tumor growth in vivo. Considering the ability of E-cadherin loss to lead to resistance to immune checkpoint blockade, it could provide insight into how immunotherapeutic approaches that block TGF-β could enhance responses and overcome resistance to immunotherapy [129].

## 5. Conclusions

The discovery of ICIs has driven significant improvements in immunotherapy. ICIs are effective in both adjuvant and primary treatment settings, particularly for melanoma, which has high immunogenicity. Despite playing a significant role in various stages of cancer treatment, a number of patients have acquired resistance to ICIs and failed to benefit from treatment. Therefore, to achieve more effective treatment outcomes, it is essential to elucidate the mechanisms that contribute to drug-resistance to ICIs.

In this review, we included many studies that examined resistance mechanisms in melanoma. However, most of them primarily focused on cutaneous melanoma. Mucosal melanoma, including OMM, is relatively rare, comprising a smaller percentage of melanoma diagnoses, which resulted in limited available data for study. Despite this situation, cutaneous melanoma, which has been studied more extensively, may offer valuable insights and serve as a reference point for understanding resistance in OMM. Thus, our inclusion of such studies aimed to bridge the knowledge gap and provide a broader perspective on immunotherapy resistance in melanoma.

Due to the shared origin of melanomas, some resistance mechanisms may also be shared between cutaneous and mucosal melanoma. However, it is important to note that OMM exhibits unique clinical and molecular characteristics that could give rise to resistance mechanisms not observed in cutaneous melanoma. Overall, it is important to thoroughly elucidate the factors contributing to resistance to ICI therapy and develop effective combination treatments aimed at overcoming these obstacles.

## Figures and Tables

**Table 1:** Ongoing clinical trials of immune checkpoint inhibitors for mucosal melanoma.

Clinical Trial İdentifier	Trıal Name	Phase	Location(s)	Status	Target Disease	Target Accrual	Intervention	Primary Outcome Measures	Secondary Outcome Measures
NCT03178123	A phase II Randomized, Control, Multi-center Study of Recombinant Humanized Anti-PD-1 mAb For Injection Compared to High-dose Interferon in Patients with Mucosal Melanoma That Has Been Removed by Surgery	Phase II	Beijing Cancer Hospital	ACTIVE, NOT RECRUITING	Mucosal melanoma that has been removed by surgery	220	Humanized anti-PD-1 monoclonal antibody toripalimab or high-dose recombinant interferon a-2B	Recurrence-free survival time (RFS)	Distant metastasis-free survival, recurrence—free survival rate at 3 years, overall survival (OS), number of participants with treatment-related adverse events
NCT05384496	Phase 2 Study of Axitinib + PD-1 Blockade in Mucosal Melanoma With Pilot Addition of Stereotactic Body Radiotherapy or Ipilimumab in Select Progressors	Phase II	Memorial Sloan Kettering Cancer Center USA	RECRUITING	Advanced or metastatic mucosal melanoma that has not been treated before	20	Combination of axitinib and nivolumab with or without SBRT/Ipilimumab	Best objective response	–
NCT05169957	Hepatic Ablation of Melanoma Metastases to Enhance Immunotherapy Response, a Phase I Clinical Trial (HAMMER I)	Phase I	University of Michigan Rogel Cancer Center, USA	RECRUITING	Metastatic melanoma with liver metastases who are at significant risk of not benefiting from systemic therapy alone	18	Ipilimumab + Nivolumab + Stereotactic Body Radiation Therapy (SBRT)	Percentage of patients who receive all planned radiotherapy.	Proportion of patients who develop grade 3 or higher toxicity, OS, PFS, proportion of patients with local control, objective response rate, BoR
NCT05089370	Oral Decitabine/Cedazuridine (DEC-C) in Combination With Nivolumab as a Strategy to Enhance the Efficacy of Immune Checkpoint Blockade in Unresectable or Metastatic Mucosal Melanoma	Phase Ib/II	University of Colorado Hospital, USA	RECRUITING	Unresectable or metastatic mucosal melanoma	30	Oral decitabine/cedazuridine (DEC-C) in combination with nivolumab	Safety of DEC-C in combination with nivolumab	The response rate to DEC-C in combination with nivolumab, to determine if the addition of DEC-C to nivolumab increases PFS and OS
NCT05111574	A Randomized Phase II Trial of Adjuvant Nivolumab With or Without Cabozantinib in Patients with Resected Mucosal Melanoma	Phase II	Multicenter, USA, CANADA	SUSPENDED	Mucosal melanoma	99	Nivolumab and cabozantinib or nivolumab and placebo	RFS	OS, RFS, PFS, overall response rate, duration of response, incidence of adverse events
NCT03033576	A Phase II Randomized Study of Nivolumab (NSC-748726) With Ipilimumab (NSC-732442) or Ipilimumab Alone in Advanced Melanoma Patients Refractory to an Anti-PD1 or Anti-PD-L1 Agent	Phase II	Multicenter, USA	ACTIVE, NOT RECRUITING	Unresectable stage IV or stage III melanoma and refractory to an anti-PD1 or anti-PD-L1 agent	94	Ipilimumab or nivolumab and ipilimumab	Progression free survival	Change in CD8^+^ expression, overall objective response rate, OS, number of participants with Gr 3 through 5 adverse events that are related to study drugs
NCT03235245	Combination of Targeted Therapy (Encorafenib and Binimetinib) Followed by Combination of Immunotherapy (Ipilimumab and Nivolumab) vs Immediate Combination of Immunotherapy in Patients With Unresectable or Metastatic Melanoma With *BRAF* V600 Mutation: an EORTC Randomized Phase II Study (EBIN)	Phase II	Multicenter, International	ACTIVE, NOT RECRUITING	*BRAF* V600 mutation-positive stage III or IV cutaneous or mucosal melanoma	271	Nivolumab + Ipilimumab, or Encorafenib + Binimetinib + Nivolumab + Ipilimumab	Progression free survival	OS, CR rate, time to CR, duration of CR, best overall response rate, time to best response, duration of best response, occurrence of adverse events, PFS2
NCT04511013	A Randomized Phase 2 Trial of Encorafenib + Binimetinib + Nivolumab vs Ipilimumab + Nivolumab in *BRAF*-V600 Mutant Melanoma With Brain Metastases	Phase II	Multicenter, USA	RECRUITING	*BRAF*-V600 mutant melanoma that has spread to the brain	112	Encorafenib+ Binimetini+ Nivolumab or Nivolumab+ Ipilimumab	Progression-free survival	OS, ICRR, objective response rate, duration of response
NCT05341349	Safety and Efficacy of SRS and Immune Checkpoint Inhibitors (ICI) Concurrent With NovoTTF-100M in Melanoma Brain Metastases	Phase I	Emory University Hospital/Winship Cancer Institute, USA	RECRUITING	Melanoma with brain metastasis	10	SRS, pembrolizumab, TTFields or nivolumab, ipilimumab, SRS, TTFields	The percentage of patients developing grade 3 CNS toxicity	Rates of skin toxicity, rates of alopecia, time to progression, intracranial control, PFS, OS
NCT05545969	A Multicentre, Open Label, Phase II Study to Determine the Response to Neoadjuvant Pembrolizumab and Lenvatinib Followed by Adjuvant Treatment With Pembrolizumab and Lenvatinib in Mucosal Melanoma	Phase II	Multicenter, Australia	NOT YET RECRUITING	Fully-resectable mucosal melanoma	44	Neoadjuvant pembrolizumab & Lenvatinib + definitive surgery + adjuvant pembrolizumab	Change in immune cell expression of HIF1 and immune cell densities, pathological response rate	RECIST response rate
NCT05436990	A Phase II Multicenter Study to Assess the Antitumor Activity of Vactosertib in Combination With Pembrolizumab in Acral and Mucosal Melanoma Patients Progressed From Prior Immune Check Point Inhibitor	Phase II	Multicenter, Severance Hospital, Yonsei University Healthy System	NOT YET RECRUITING	Acral or mucosal melanoma with stage IV or unresectable stage III disease, progressed on treatment with an anti-PD-1/L1 mAb	30	Vactosertib in combination with pembrolizumab	Overall response rate	–
NCT05420324	A Multicenter, Single-arm, Open-label Phase II Study to Evaluate the Efficacy and Safety of YH003 in Combination With Pebolizumab and Albumin Paclitaxel in First-line Treatment of Patients With Unresectable/Metastatic Mucosal Melanoma	Phase II	Multicenter, China	RECRUITING	Advanced or cytologically confirmed metastatic or unresectable mucosal melanoma	43	YH003 in combination with pebolizumab and albumin paclitaxel in first-line treatment	Confirmed objective response rate	–
NCT03313206	Phase II Multicentric Study: Efficacy Evaluation of Neoadjuvant Treatment Associated With Maintenance Therapy by Anti-PD1 Immunotherapy on Disease-free-survival (DFS) in Patients With Resectable Head and Neck Mucosal Melanoma	Phase II	Multicenter, France	RECRUITING	Resectable head and neck mucosal melanoma.	60	Neoadjuvant pembrolizumab + Surgery + IMRT or neoadjuvant pembrolizumab + Surgery + IMRT + Lenvatinib	Disease free survival	–
NCT04622566	A Phase II Study of Neoadjuvant Lenvatinib and Pembrolizumab in Resectable Mucosal Melanoma	Phase II	Beijing Cancer Hospital	NOT YET RECRUITING	Resectable mucosal melanoma	26	Lenvatinib and pembrolizumab	Pathological complete response (pCR) rate	1 year RFS rate per RECIST1.1 as assessed by investigator, OS, incidence of AEs/SAEs
NCT04318717	Adjuvant Pembrolizumab and Hypofractionated Radiation Therapy for the Treatment of Mucosal Melanoma	Phase II	Washington University School of Medicine	RECRUITING	Mucosal melanoma that has undergone surgical resection	16	Pembrolizumab + Hypofractionated radiation therapy	Local tumor control rate	Number of treatment-related grade 3 or greater adverse events, number of treatment discontinuations due to treatment-related adverse events, RFS, distant metastasis-free survival, OS
NCT02506153	A Phase III Randomized Trial Comparing Physician/Patient Choice of Either High Dose Interferon or Ipilimumab to MK-3475 (Pembrolizumab) in Patients With High Risk Resected Melanoma	Phase III	Multicenter, USA, CANADA, IRELAND	ACTIVE, NOT YET RECRUITING	Stage IIIA (N2a), IIIB, IIIC, or stage IV melanoma	1378	High-dose recombinant interferon alfa-2B, ipilimumab or pembrolizumab	OS, RFS, PD-L1 status	Incidence of toxicity, post-relapse therapy, *BRAF* mutation status, long-term survival, change in quality of life
NCT03698019	A Phase II Randomized Study of Adjuvant Versus NeoAdjuvant Pembrolizumab (MK-3475) for Clinically Detectable Stage III-IV High-Risk Melanoma	Phase II	Multicenter, USA	ACTIVE, NOT YET RECRUITING	Detectable stage III (clinically detectable N1b, N1c, N2b, N2c, N3b and N3c) or stage IV resectable melanoma	323	Adjuvant pembrolizumab or adjuvant and neoadjuvant pembrolizumab	Two-year event-free survival rate	Number of participants with grade 3 through 5 adverse events that are related to study drug, two-year OS rate, response rate, number of participants receiving surgery
NCT05549297	Phase 2/3 Randomized Study of Tebentafusp as Monotherapy and in Combination With Pembrolizumab Versus Investigator’s Choice in HLA-A*02:01-positive Participants With Previously Treated Advanced Melanoma (TEBE-AM)	Phase II/III	Multicenter, USA	RECRUITING	Unresectable Stage III or stage IV non-ocular melanoma	460	Tebentafusp or tebentafusp in combination with pembrolizumab or investigators choice of therapy	ctDNA reduction on treatment relative to baseline, OS	Safety; adverse events and severe adverse events, safety: tolerability, serum pharmacokinetics, incidence of anti-tebentafusp antibodies
NCT03241186	Single Arm Phase II Study of Ipilimumab and Nivolumab as Adjuvant Therapy for Resected Mucosal Melanoma (SALVO Study). HCRN: MEL16-252	Phase II	Multicenter, USA	ACTIVE, NOT YET RECRUITING	Melanoma of any mucosal site, resected within ≤90 days of registration.	36	Ipilimumab (1 mg/kg) + Nivolumab (3 mg/kg) IV	Recurrence-free survival time	Adverse Events, OS
NCT04091217	Atezolizumab in Combination With Bevacizumab in Patients With Unresectable Locally Advanced or Metastatic Mucosal Melanoma	Phase II	Multicenter, China	COMPLETED	Unresectable locally advanced(stage III) or metastatic(Stage IV) mucosal melanoma	43	Atezolizumab + Bevacizumab	Objective response rate	PFS, OS, DOR, DCR, percentage of participants with adverse events

Abbreviations: OS, Overall survival; PFS, Progression free-survival; BoR, Best overall response; MM, Mucosal melanoma; CR, Complete response; PFS2, Progression-free survival 2; DOR, Duration of objective response; DCR, Disease control rate; ICRR, Intracranial response rate; SRS, Stereotactic Radiosurgery; TTFields, Tumor Treating Fields Therapy.

## Data Availability

The data presented in this study are available within this article.
